# Near-infrared hyperspectral imaging and robust statistics for *in vivo* non-melanoma skin cancer and actinic keratosis characterisation

**DOI:** 10.1371/journal.pone.0300400

**Published:** 2024-04-25

**Authors:** Lloyd A. Courtenay, Innes Barbero-García, Saray Martínez-Lastras, Susana Del Pozo, Miriam Corral de la Calle, Alonso Garrido, Diego Guerrero-Sevilla, David Hernandez-Lopez, Diego González-Aguilera

**Affiliations:** 1 CNRS, PACEA UMR 5199, Université de Bordeaux, Bât B2, Pessac, 33600, France; 2 Department of Cartographic and Land Engineering, Higher Polytechnic School of Ávila, Universidad de Salamanca, Ávila, Spain; 3 Dermatology Service, Ávila Healthcare Complex, Ávila, Spain; 4 Institute of Regional Development, University of Castilla la Mancha, Campus Universitario s/n, Albacete, Spain; Memorial Sloan Kettering Cancer Center, UNITED STATES

## Abstract

One of the most common forms of cancer in fair skinned populations is Non-Melanoma Skin Cancer (NMSC), which primarily consists of Basal Cell Carcinoma (BCC), and cutaneous Squamous Cell Carcinoma (SCC). Detecting NMSC early can significantly improve treatment outcomes and reduce medical costs. Similarly, Actinic Keratosis (AK) is a common skin condition that, if left untreated, can develop into more serious conditions, such as SCC. Hyperspectral imagery is at the forefront of research to develop non-invasive techniques for the study and characterisation of skin lesions. This study aims to investigate the potential of near-infrared hyperspectral imagery in the study and identification of BCC, SCC and AK samples in comparison with healthy skin. Here we use a pushbroom hyperspectral camera with a spectral range of ≈ 900 to 1600 nm for the study of these lesions. For this purpose, an ad hoc platform was developed to facilitate image acquisition. This study employed robust statistical methods for the identification of an optimal spectral window where the different samples could be differentiated. To examine these datasets, we first tested for the homogeneity of sample distributions. Depending on these results, either traditional or robust descriptive metrics were used. This was then followed by tests concerning the homoscedasticity, and finally multivariate comparisons of sample variance. The analysis revealed that the spectral regions between 900.66–1085.38 nm, 1109.06–1208.53 nm, 1236.95–1322.21 nm, and 1383.79–1454.83 nm showed the highest differences in this regard, with <1% probability of these observations being a Type I statistical error. Our findings demonstrate that hyperspectral imagery in the near-infrared spectrum is a valuable tool for analyzing, diagnosing, and evaluating non-melanoma skin lesions, contributing significantly to skin cancer research.

## 1. Introduction

Among all types of malignancies, Non-Melanoma Skin Cancer (NMSC) is the most common type among Caucasian populations [1]. NMSC mostly affects elder populations, and is typically found on areas of skin with high exposure to sun-light [2]. The incidence of NMSC is constantly increasing [3,4], while the rise in cases is expected to continue, mostly as a consequence of increased sun exposure as well as other demographical phenomenon [5]. From this perspective, early detection of NMSC is highly important so as to reduce treatment complexity, while regulating the impact this pathology may have on health services [6,7].

The main types of NMSC are Basal Cell Carcinoma (BCC) and cutaneous Squamous Cell Carcinoma (SCC, or cSCC), which together, represent approximately 99% of all NMSCs. BCC is the most common type, representing up to 85% of cases [6]. Other types of NMSC include Merkell-Cell carcinomas, cutaneous lymphomas, adnexal tumours and other primary cutaneous neoplasms [8]. BCC and SCC have important differences; SCC is capable of metastasis and can be fatal in a subset of cases [9], while BCC has almost no metastasis risk [7].

Similarly, Actinic keratosis (AK) is an extremely common skin lesion caused mainly due to sun exposure. AKs are often treated as premalignant lesions, as they are considered to represent an early stage of SCC [10–12]. A range between 0.025% and 16% [13,14] of all AKs progress into SCC. Studies have shown a link between AK and higher risk of skin cancer, including SCC, BCC, and malignant melanoma. For these reasons, research into the detection of AK can also be considered important to reduce the incidence of skin cancer [15], and monitor these patients to allow early diagnosis if the lesion’s condition deteriorates.

The most common means of diagnosing and detecting skin cancer detection is through visual examination, in many cases aided through the use of dermoscopy. The subjectivity of visual biomarkers, however, compromises the accuracy of visual diagnosis [16]. Dermoscopy is a non-invasive *in vivo* technique that makes use of polarized light, a microscope, and liquid immersion, to be able to analyze and diagnose skin lesions. While the dermoscopic features of different NMSC are well defined [17], issues often arise discerning between cases of AK with SCC [18]. Likewise, while dermoscopy improves the accuracy of naked-eye visual inspection, it requires a high level of training, and consensus between more than one experts is advised [19]. Finally, the most accurate means of diagnosing skin lesions is through biopsy, followed by histopathological analyses [18]. This approach, however, are highly invasive and often costly.

Several methodologies have been proposed over the years for the development of a non-invasive and accurate tool for *in vivo* detection and characterization of skin lesions such as NMSC and AK. Among them, Hyperspectral Imagery (HSI) is one of the most promising approaches. Nevertheless, while HSI is an accurate tool, a lot of research is still required to find the most efficient and precise means of using this type of sensor, while the majority of existing research is focused on the study of melanoma, as opposed to NMSC. HSI is capable of obtaining the spectral response of tissue in a large number of different wavelengths, usually beyond the visual range of the electromagnetic spectrum. It combines the spectral resolution of spectroscopy with the spatial resolution of images.

**Fig 1:**
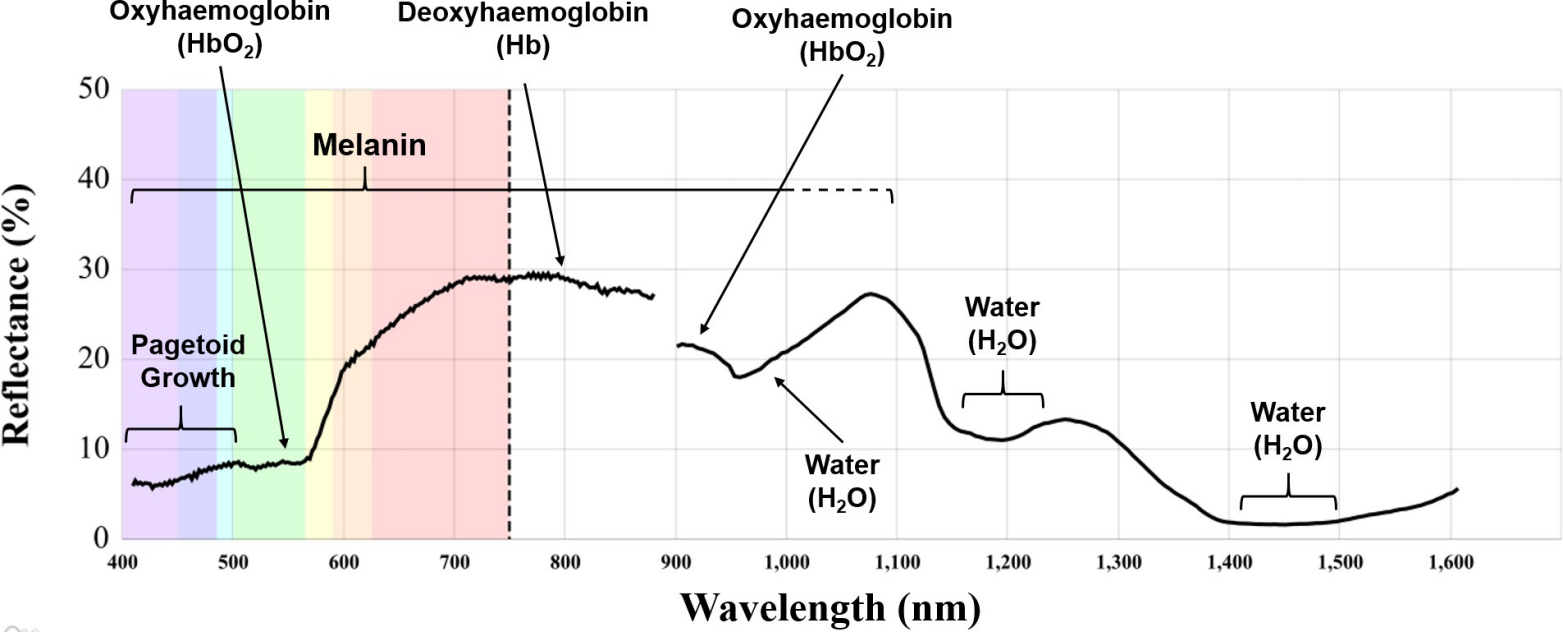
Visible and near-infrared spectrum of skin samples with indications of conditioning factors in light absorption at specific regions of the electromagnetic spectrum. The curve presented in the visible region of the spectrum was obtained by data from Courtenay et al., [25], while the curve represented beyond 900 nm is data from the present study.

Existing studies using HSI for this purpose typically focus on wavelengths in the Visible to Near-Infrared (VNIR: 400–1000 nm), or Near-Infrared (NIR: 800–2500 nm), region of the electromagnetic spectrum. The value of analysing skin beyond the range of the visible spectrum is the extended range of penetration IR light has in biological tissue [20]. IR light can therefore be used to assess the properties of cancerous and healthy tissue, considering features like; water content and saturation, lipid content, collagen levels, deoxyhaemoglobin and oxyhaemoglobin ratios, different protein structures, as well as the chemical composition of certain elements of skin [21–23] (Fig 1).

For general purposes, two main windows have been identified for the analysis of biological tissues, ranging between (1) 650–950 nm and (2) 1000–1350 nm. The latter is less studied due to the higher cost of sensors and illumination devices in this wavelength range [20]. Studies show that VNIR ranges between 450–950 nm are especially useful for the detection of pigmented skin lesions [24], while promising results have also been obtained for the particular case of NMSC [25,26]. Nevertheless, other studies focus on larger wavelengths such as 900–1500 nm [27] or wider ranges including the visual part of the spectrum and the NIR with 370–1600 nm using spectroscopy on *ex vivo* samples [28,29]. These studies suggest divergences between healthy and cancerous skin (specially non-melanoma) for the range 1050–1400 nm, as NMSC have lower absorption rates for certain tissues in comparison with healthy skin [28]. Likewise, few studies assess the differences between AK and NMSCs from this perspective, which could be considered a valuable tool to the development of diagnostic tools.

The present study develops on this research by exploring the robust statistical patterns of hyperspectral signatures obtained from samples of BCC, SCC, AK, and healthy skin. From this, our objectives, therefore, are to research ways in which NIR based HSI can shed a light on the differentiation between these different types of NMSCs. For this purpose, this study employs the use of a pushbroom hyperspectral linear camera registering wavelengths between 900 and 1700 nm, over the course of 166 spectral bands. Using robust statistics, the degree of separation between tissue samples is assessed, so as to define an optimal spectral window for the characterization and analysis of these types of lesions. Building from this, future research using complex computer vision and artificially intelligent algorithms can be built upon for screening tools in early NMSC and AK diagnosis.

## 2. Materials and methods

This study employs a pushbroom hypsepectral imaging sensor to analyze the spectral properties of patients with BCC, SCC, and AK. First we detail the design of an *ad hoc* platform created for image acquisition, featuring a motorized platform with controlled illumination. Calibration methods are outlined, followed by the photographic documentation of suspicious skin lesions, subsequently diagnosed histopathologically. Rigorous data analysis involves statistical tests for the homogeneity of sample distributions, adjusting subsequent analyses accordingly. Our methodological approach intends to use this workflow so as to identify a specific electromagnetic spectrum window more likely to reveal statistical differences between healthy skin and each type of skin lesion.

### 2.1. Sensor and setup

A Headwall Hyperspec NIR X Series hyperspectral imaging sensor, with an 8 mm focal lengthand a wavelength range of 900–1700 nm, was used for the acquisition of the hyperspectral images (Table 1). This camera is composed of a pushbroom sensor, which captures a linear array of pixels (1 x 320 px), building on the approach presented by Courtenay et al. [25] for image acquisition.

**Table 1:** Specifications for the hyperspectral pushbroom camera, Headwall Hyperspec NIR X Series, used for the present study.

	Headwall Hyperspec NIR X Series
Image Modality	Pushbroom
Sensor Type	InGaAs
Number of Spectral Bands	166
Wavelength Range	900–1700 nm
Space between Spectral Bands	4.8 nm
Spatial Resolution	320 px
Pixel Size	30 μm
Focal Length	8 mm
Sensor Size	9.6 mm
Weight	2.9 kg
Platform Push-speed	45 mm/s
Full-Width at Half Maximum	4 nm
Entrance Slit Width	25 μm
Bit-depth	14-bit

**Fig 2:**
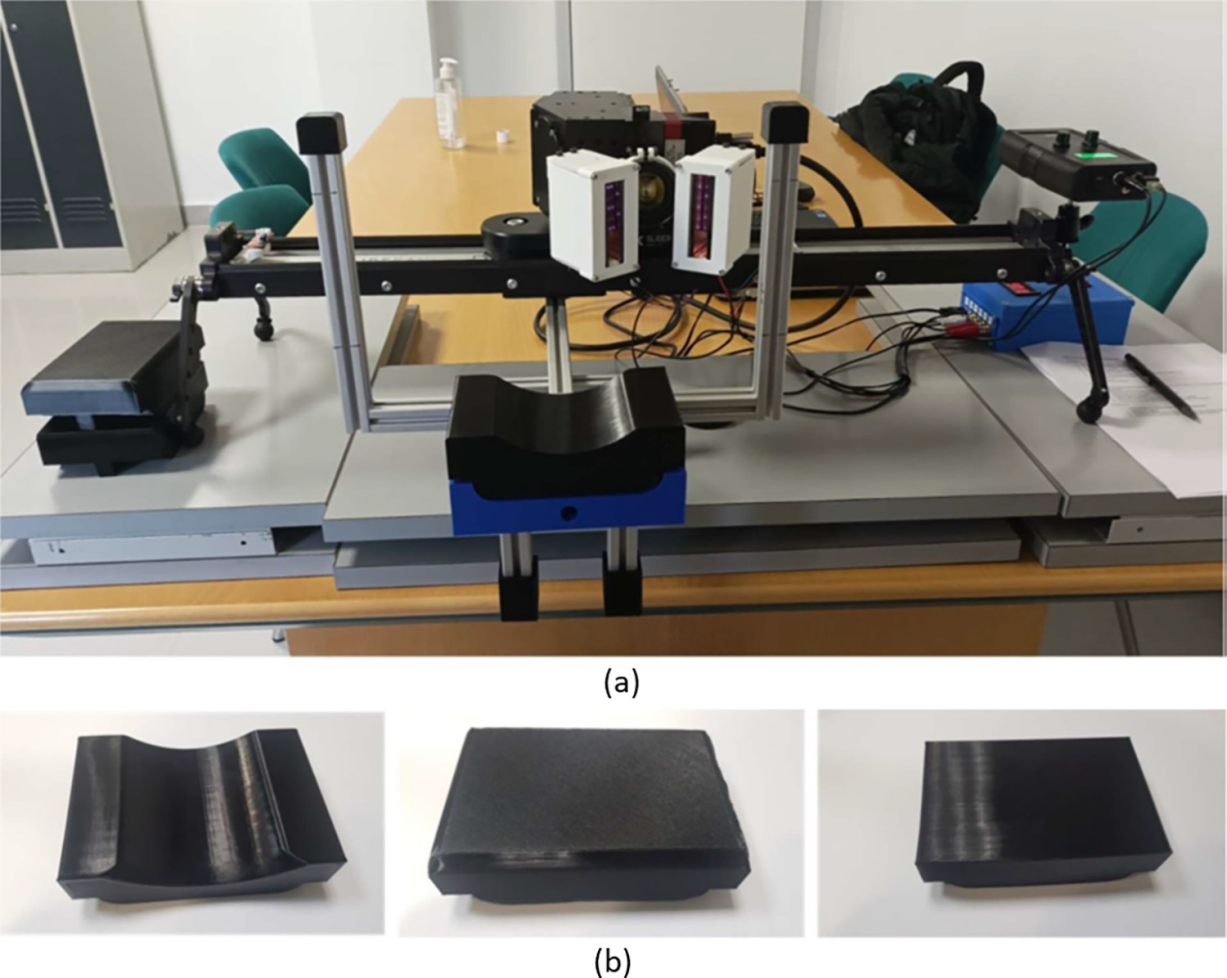
(a) The hyperspectral pushbroom platform built for the purpose of data acquisition in the present study. (b) The different support platforms to help with patient comfort during the acquisition process.

While pushbroom cameras are highly popular sensors in HSI, with higher spatial resolution as opposed to other types of sensors, they present the distinct disadvantage of requiring the sensor to be moved, or *pushed*, along the *x*-axis in order to obtain an entire image of more than one column of pixels. In order to perform this, an *ad hoc* platform was designed, following the proposals by Courtenay et al. [25]. This system consists of the camera itself, a motorized base, an illumination system, a controller, as well as a laptop (Fig 2). Modifications were made, however, to adapt the system to dimensions, optical displacement range, and speed conditions, of the new sensor.

Considering observations made by Courtenay et al. [25], regarding the comfort of the patient during image acquisition, however, a support was included so that patients could rest or lean their weight on (Fig 2A) during the process of taking each photo (the image acquisition takes approximately ≈11 seconds). This consists of a rigid metallic beam, attached to a base where a series of different 3D printed resting platforms could be attached. These platforms were printed with a semi-hollow body, so that the overall platform was flexible and softer than a standard rigid support system. Platforms were additionally printed with three different shapes that were moulded to support different parts of the body where skin lesions may be present; a curved surface where a patient’s chin or arm could rest; as well as two different types of flat surfaces for the support of other body parts such as hands and arms. In cases where lesions were presented on the back or chest of the patient, the support system could be detached allowing the patient to come closer to sensor. This modification to the system presented by Courtenay et al. [25] was found to be especially important when working with elderly patients, who may struggle to keep still or in position during the image acquisition process.

The illumination system used was composed of two arrays of 21 LEDs. Seven different LEDs were used, with peak wavelengths of 880, 940, 1050, 1200, 1300, 1450, and 1550 nm. The overlap of these wavelengths assures that almost the entire operating range of the camera is covered. The LEDs and Printed Circuit Board were wrapped with four parallel mirrors, leaving as output a band through which the lighting leaves the system with hardly any losses. These mirrors are top-notch reflection and reflect about 96% of the light in the 700–2000 nm band. The system of illumination was adjusted and attached to the camera lens by use of an *ad hoc* designed ring. The position of the system was optimized to avoid shaded areas.

Both the platform and the camera were controlled using an external electronic module device, synchronizing platform movement, activation of the illumination system, as well as the sensor’s shutter speed. The camera was additionally controlled using the HyperSpec III software.

### 2.2. Data acquisition

Image acquisition was carried out by expert technicians at the Ávila Healthcare Complex (Ávila, Spain), clinic and hospital.

Sensor calibration was carried out using HyperSpec III. The calibration was a semiautomatic process, involving capturing images of a white and dark reference standards so as to convert raw uncorrected data (*S*) from images, to tensors (*X*) containing reflectance values (%) of each wavelength of light (Eq 1). Dark pixel offset (*B*) was taken with the lens cap on, while white reference standards (*W*) were taken using a known reflectance pattern (Spectralon). This calibration process was performed using the HyperSpec III software, and following the mathematical protocol defined by Geladi et al. [30];

$$X=100(S-B){\left(W-B\right)}^{-1}$$ (1)

The final height of the acquired images was 190 px, with a width of ≈650 px, the reduction in comparison with the camera spatial resolution (Table 1) was required to limit the images to the illuminated area while maintaining the lesions at an optimal distance from the camera. Likewise, due to illumination constraints, not all the bands were found to be useful, therefore the pixels captured only contained 150 of the 166 bands captured by the camera. The final wavelength range captured in this study was calculated to be 900–1606 nm.

Once images had been obtained, both the technicians and dermatologists collected and verified all patient information, including suspected diagnosis, age, gender, location of the lesion, and time of evolution. In this first phase, lesions were visually identified by dermatology experts. After taking images, a biopsy and histopathology were performed for each lesion to confirm diagnosis.

### 2.3. Sample

A total of 125 patients with observed skin lesions (66 with BCC, 42 with SCC, and 17 with AK) were studied with the hyperspectral sensor, during the months of March to July, 2022. During data acquisition, technicians were required to check each image for sufficient quality, and were required to re-capture images if lesions could not be clearly identified in the image. For this reason, no further quality control was required and no images were discarded from the initial sample. Patients included within this study were majority fair skinned, while ages ranged from 23 to 99 years of age, with a median age of 79, and asymmetric 95% confidence intervals ranging from 47 to 93. Of this sample, 40.5% of the patients were female, and 59.5% were male.

All patients agreed to participate in the study; however, due to patient animosity and data protection, no further details have been disclosed. Patients were provided with informed consent via a signed written document.

The study was conducted in accordance with the Declaration of Helsinki and approved by the Ethics Committee of Investigation with Medicine in the Health Sector of Ávila (02/2022, SA097P20).

### 2.4. Data processing

From each lesion, 30 pixels containing hyperspectral signatures were obtained, depending on the size and clarity of lesion boundaries. Another 30 pixels of healthy skin were also selected, obtaining pixels from points that were as far away from the tumour as possible, so as to avoid possible contamination. For this propose, regions of interest ROI were manually delimited followed by the random sampling of 30 pixels from each area. This process was automated using the Python (v.3.7) programming language. The final number of spectral signatures obtained were: 4,500 for Healthy (H) samples, 1,980 for BCC samples, 1,260 for SCC samples, and 510 for AK samples (total *n* = 8,250 signatures).

A statistical power analysis for each of these sample sizes according to Cohen’s δ [31] was calculated at <0.99, with an α value of 0.003 and an effect size (δ) values of 0.8. Even for smaller δ values (e.g., δ = 0.2), these calculations showed that even for the smallest sample (AK, *n* = 510), the present sample has a 93.7% probability of detecting an alternative hypothesis, thus supporting our given sample sizes. From a similar perspective, given the present dataset describes samples with a total of 150 variables (i.e., bands), variable to sample size ratios are still low enough to be considered acceptable (≪ 1; [32]), with the highest values for the lowest sample size calculated at 0.29, with other ratios being calculated as low as 0.03. Finally, considering the skew in sample sizes, with AK being a much smaller sample size than H, the overall balance (*B*, Eq 2; adapted from [33,34]) of the dataset has been calculated at 0.82 (i.e., a moderate imbalance is present in the dataset when considering all samples together). *B* was calculated using:

$$B=\frac{-\sum_{i=1}^{g}\frac{c_{i}}{n}\log\frac{c_{i}}{n}}{\log g}$$ (2)

where *c* is the count of individuals in group *g*, and the sum of all values in vector *c* is *n* (i.e., the total sample size).

Once signatures had been selected, samples were tested for normality using Shapiro-Wilks tests [35]. After that, visual inspection of density plots and quantile-quantile plot was carried out [36]. If samples were found to fit a Gaussian distribution, then subsequent analyses adopted a parametric approach, while non-Gaussian distributions were studied using robust statistical methods (*sensu* [25]). To provide additional metrics to assess distribution homogeneity, kurtosis and skewness were also computed.

Once sample homogeneity was verified, different statistical approaches were used to define the hyperspectral “signature” of each sample. This approach was found to be particularly useful when considering the noisy nature of HSI data, which often presents a high number of anomalies and outliers.

For descriptive statistics, the sample mean or median were used to calculate central tendency, using the former for homogeneous and the latter for inhomogeneous distributions respectively [36–41]. Likewise, assessment of sample variance was calculated either using the standard deviation, or the Square Root of the Biweight Midvariance (√BWMV), for homogeneous and inhomogeneous distributions respectively [38,41]. Next, non-symmetric 95% confidence intervals were constructed using the [0.05, 0.95] interquantile range [36].

For univariate hypothesis testing, each wavelength for samples were then analysed for homoscedasticity via Bartlett’s parametric test [42], or Levene’s non-parametric test [43]. In each of these tests, the Null Hypothesis (*H*_*0*_) assumes samples to have equal variance.

Finally, the calculation of multivariate differences was calculated either using Multivariate Analysis of Variance (MANOVA) tests, using the parametric Hotelling-Lawley test statistic [44], or the non-parametric pairwise Wilcoxon test [45]. Both the Hotelling-Lawley and Wilcoxon tests assume *H*_*0*_ samples to be similar. This was accompanied by the multivariate ordination of data by means of a robust kernel-Principal Component Analysis (k-PCA). k-PCA performs a non-linear transformation of data prior to the computation of a traditional PCA. This is particularly useful when dealing with highly complex and noisy data, that does not necessarily fulfil underlying assumptions of linearity or statistical normality. The kernel function (*K(x)*) considers the spatial proximity of each data point (*x*) with the rest of data (*X*), so as to provide a density-aware non-linear transformation. For the purpose of this study a spline kernel was used (Eq 3) [46];

$$K(x_{i},X_{:,n})=1+X_{:,n}x_{i}+X_{:,n}x_{i}\min(X_{:,n},x_{i})-\frac{X_{:,n}+x_{i}}{2}\min{\left(X_{:,n},x_{i}\right)}^{2}+\frac{1}{3}\min{\left(X_{:,n},x_{i}\right)}^{3}$$ (3)

All image handling applications were programmed in the Python (v.3.7.) programming language. All statistical applications were performed in the R programming language (v.4.2.0). Finally, the JavaScript programming language was used for some additional visualization tasks using the Amcharts (v.4.) library.

### 2.5. Evaluation of hypothesis test results

Taking into account the recent criticism of the "blind" use of *p-value* in applied statistics, as well as the recommendations set forth by the editors and contributors of the American Statistician, this study did not use *p* < 0.05 as a threshold for defining statistical significance [47,48]. Instead, all hypothesis testing was performed using complementary calculations of the probability of observations being a Type I statistical error, or the False Positive Risk (FPR) [49]. For FPR, the Sellke-Berger approach [50] was used for the definition of Null Hypothesis (*H*_*0*_) and Alternative Hypothesis (*H*_*a*_) likelihood ratios. Considering observations made by [25] regarding *p*-values over 0.3681, an additional evaluation of *p*-values was performed, assessing the probability of *H*_*0*_ (*P(H*_*0*_*)*). *P(H*_*0*_*)* is used as a means for calibrating any *p-*value between 0 and 1, whereby values under 0.3681 are equal to the FPR, while values above this number are calculated by the inverse of FPR [25].

In light of these calibrations, *p-values* were thus evaluated using a robust value of 0.003 (3σ) as a threshold for more conclusive results. This *p-value* can be considered to have a FPR of 4.5 +/− [1.2, 15.9] %, using priors of 0.5 +/− [0.2, 0.8] [25]. Likewise, for all calculations of both FPR and *P(H*_*0*_*)*, results were reported using prior probabilities of complete randomness (prior = 0.5), as suggested by Colquhoun [49], while confidence intervals using prior probabilities of 0.8 and 0.2 were also considered and reported.

## 3. Results

### 3.1. Characterising the hyperspectral signatures of samples

**Fig 3:**
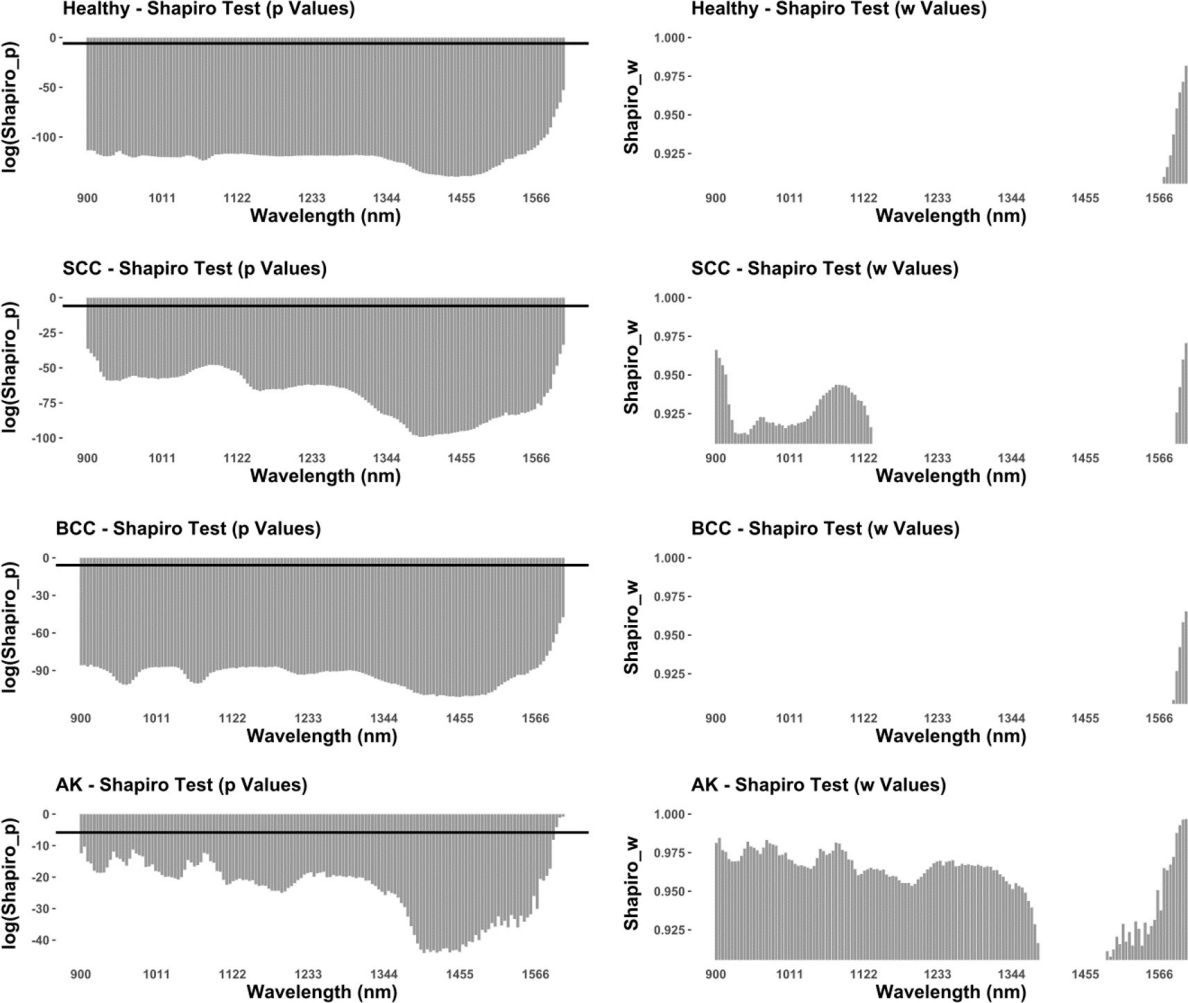
Graphs presenting the logarithm of Shaprio-Wilks p-values as well as test statistics (w) for each of the samples across each of the bands. The solid horizontal line in each of the left-hand panels mark the p = log(0.003) threshold; all log(p) values that fall below this line have less than a 5% probability of being false positives.

**Fig 4:**
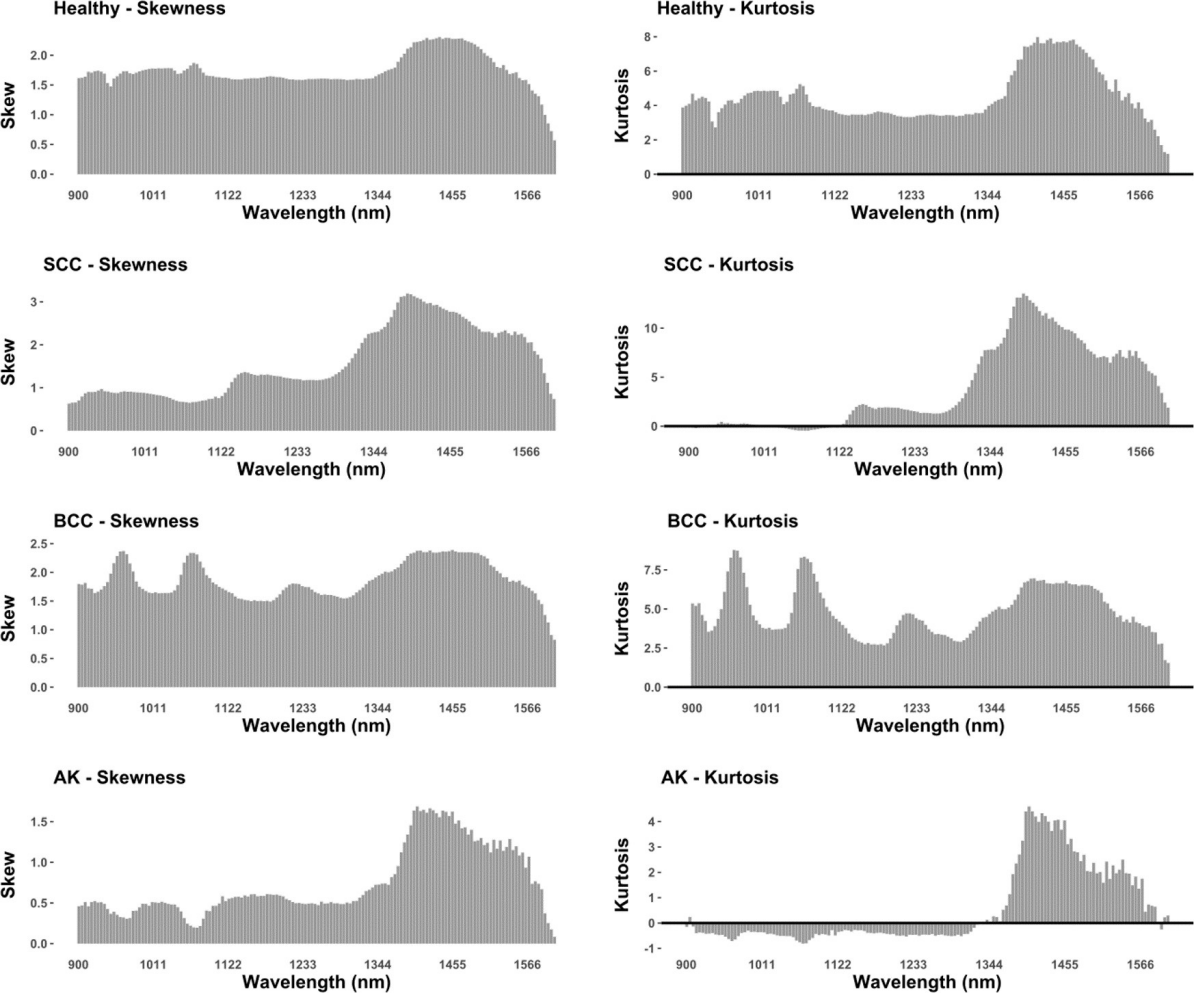
Sample skewness and kurtosis calculations for each band across the studied region of the electromagnetic spectrum.

Hyperspectral curves were found to present highly inhomogeneous distributions across all frequencies of the NIR spectrum for all 4 samples (Figs 3 and 4). In all samples, the highest probability of a False Positive, given this conclusion, is 0.015% (*w* > 0.88, *p* < 4.56e-06). From this perspective, it can be seen how all samples present high overall skewness parameters (Fig 4, skew ≪ 0.25), especially in frequencies above ≈ 1300 nm. Likewise, with the slight exception of BCC, all samples present abnormally high kurtosis values in the region of 1355.37 and 1502.20 nm (kurtosis ≈ 7.38), a region of the electromagnetic spectrum with higher absorption rates due to water content. In the case of BCC, while kurtosis values are high in this region (6.57), noticeable peaks are also present around 962.23 nm (kurtosis ≈ 8.77) and 1066.44 nm (kurtosis ≈ 8.34), with the former also being associated with areas of high absorption rates due to water saturation in skin.

**Fig 5:**
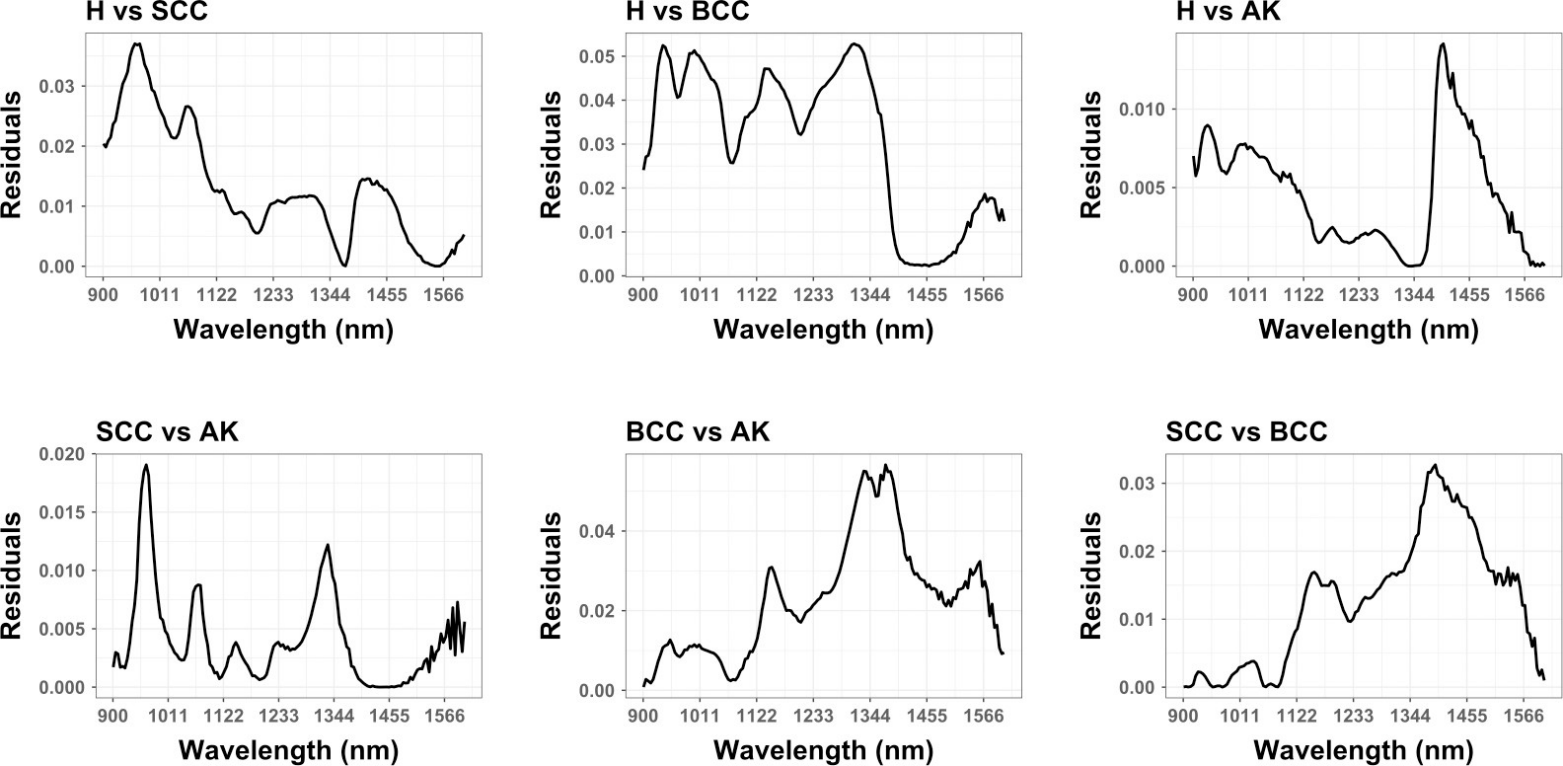
Calculated residuals for fitted linear models for the separation of different samples across the entire spectrum analysed.

Calculating residuals from linear models on this data (Fig 5) additionally highlight the existence of regions of the electromagnetic spectrum where parametric approaches are not likely to efficiently or accurately differentiate between certain samples. In many cases, these residuals are high throughout the spectrum without a particular concentration on any particular region.

In light of these observations, it can be seen how robust statistical approaches are fundamental for the analysis of data in this region of the electromagnetic spectrum, as all samples are strongly characterized by positive skew and areas of particularly high kurtosis throughout the region of 900 to 1600 nm.

**Fig 6:**
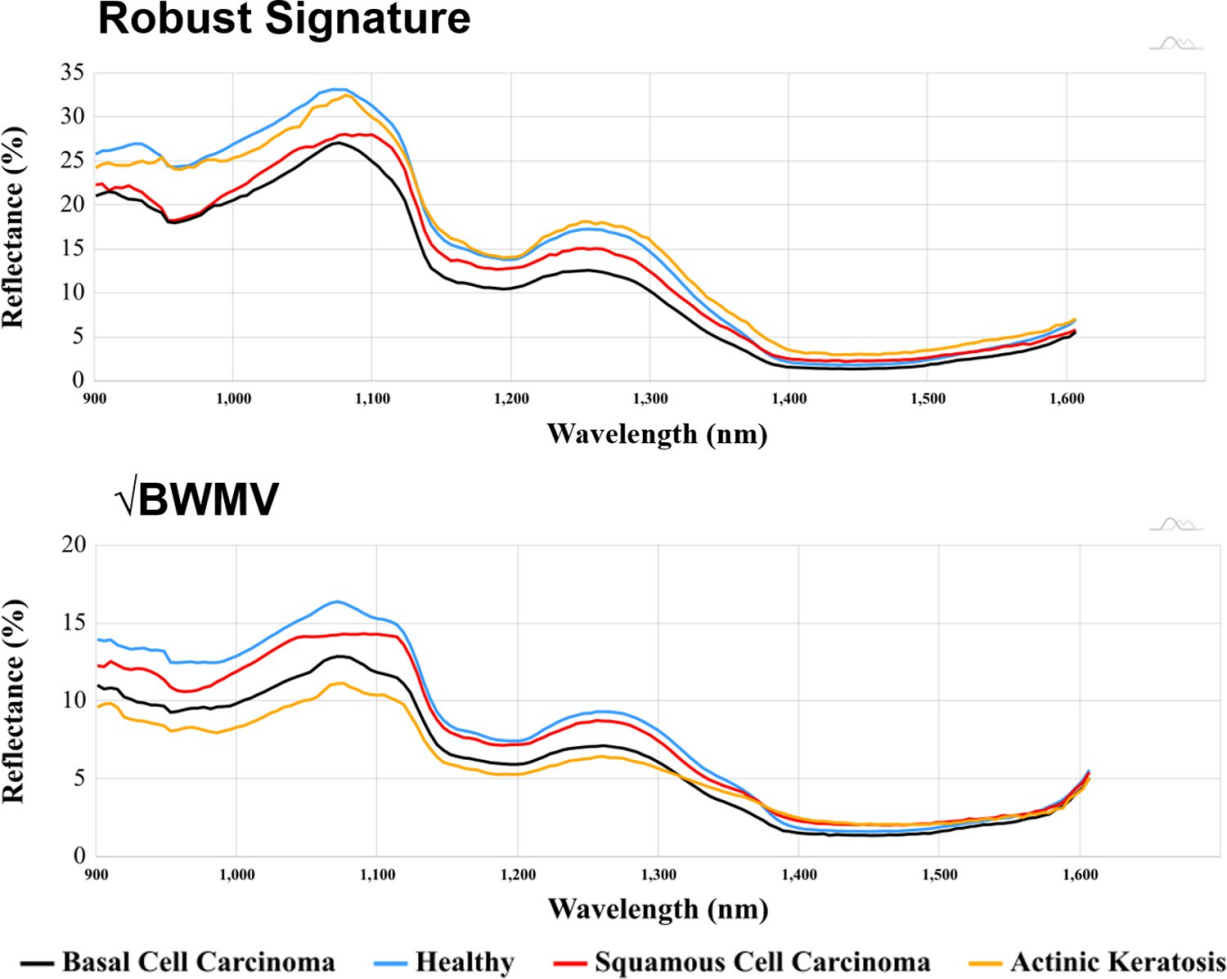
Hyperspectral signatures for each of the studies samples. Upper panel: The robust signature using the median of values for each sample a cross each wavelength. Lower panel: The robust deviation of these samples using the Square Root of the Biweight Midvariance as a measure of variation for each of the samples.

Upon plotting central tendency and variance curves (Fig 6), it can be seen how healthy skin generally reflects the most light towards the shorter wavelengths of the spectrum, in particular in regions below 1132.27 nm, while regions above 1137.48 nm present general trends for AK to reflect marginally (+0.99%) more light. Nevertheless, general calculations of sample variation (Fig 6) show H to have the potential of reflecting more light than any other sample particularly between 900.66 and 1369.58 nm (3.46% more than AK), as well as between 1540.09 and 1606.40 nm (+0.35% more than AK). From the perspective of asymmetric 95% confidence intervals, H can be seen to reflect the most amount of light across the majority of the spectrum reflecting up to 25% more light in the region between 1009.60 and 1118.54 nm. An exception is present, however, between 1388.52 and 1454.83 nm, where √BWMV values drop coinciding with an area of higher water absorption rates.

When considering both types of malignant tumours (Fig 6), both SCC and BCC are observed to absorb the most amount of light across the entire spectrum, in particular across regions below 1390 nm. From this perspective malignant cells have been observed to absorb 3.82 ∈ [1.35, 5.60]% more light than H, and 3.31 ∈ [0.17, 6.24]% more light than AK.

Analysing differences between malignant NMSC samples in further detail additionally reveal BCC to be the sample that absorbs the most amount of light across the entire spectrum (Fig 6). The areas of the spectrum where differences between BCC and SCC signatures are the lowest can be found between 948.02 and 985.91 nm, where BCC is found to generally absorb 1.73% more light than SCC samples. From the perspective of robust variance, BCC is also observed to present the lowest √BWMV values across the entire spectrum.

### 3.2. Univariate hypothesis testing

From an analytical perspective, univariate statistical testing of homoscedasticity has found a number of different windows where statistical differences can be noted in the variance between the different samples.

**Fig 7:**
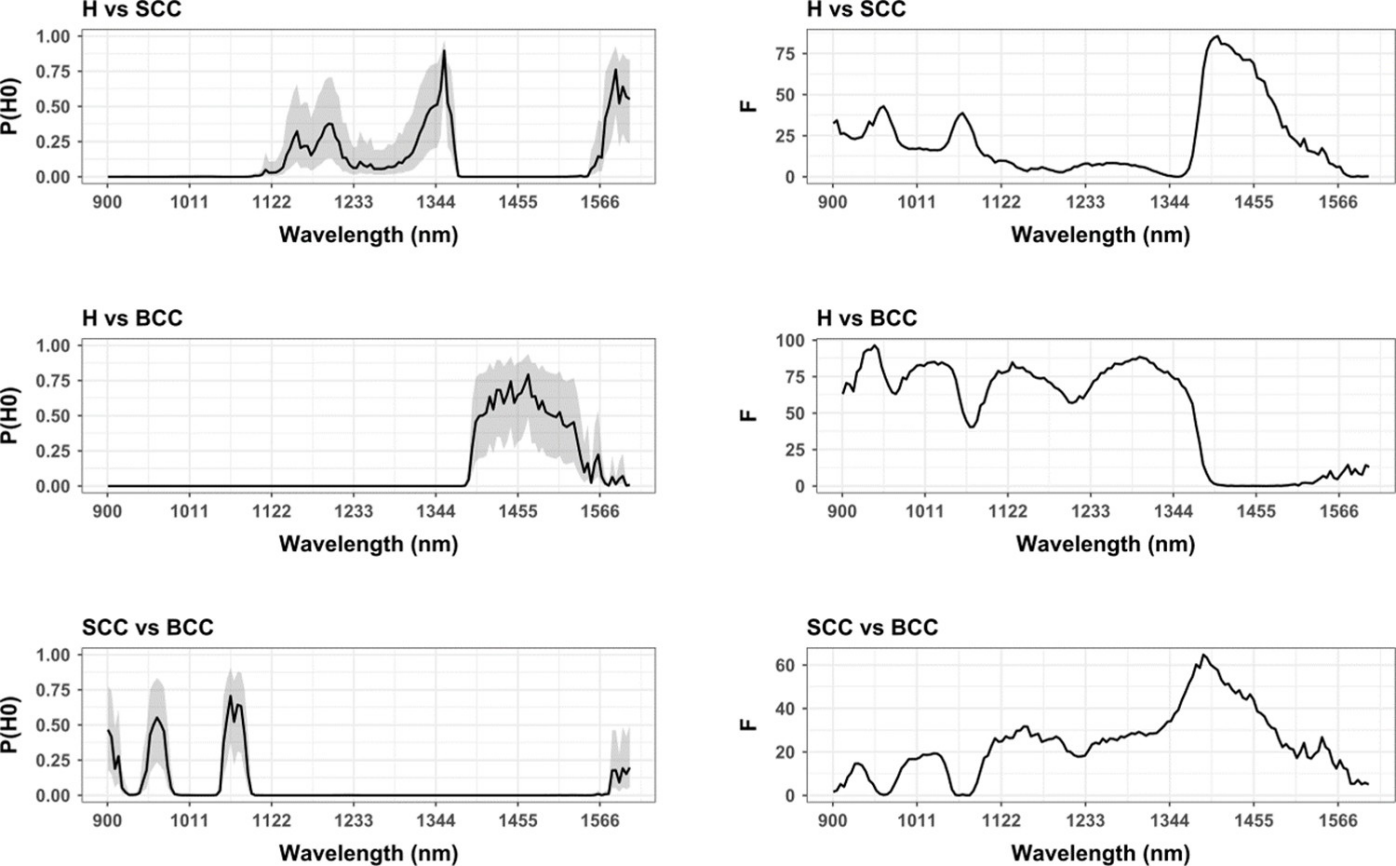
Univariate hypotheses test results comparing a number of the different samples (H, SCC and BCC) across each of the hyperspectral bands using the Levene test for homoscedasticity. (Left panels) Probability of Null Hypothesis (P(H_0_)) curves, calibrated for each p-value using priors of 1:2 to mark the central tendency, while confidence intervals mark upper bounds using 2:10 prior probabilities in favor of H_0_, and lower bounds using 8:10 prior probabilities in favor our H_0_. (Right panels) Test statistic calculations for each of the corresponding hypothesis tests.

When comparing healthy skin with both types of malignant skin tumours (Fig 7), particularly notable differences can be found across the electromagnetic spectrum represented by wavelengths shorter than 1085.38 nm. In these cases, H and SCC can be calculated to present notable divergences (Fig 7; central *F* = 25.1, *p* = 5.68 ×10^−07^), while H and BCC can also be noted to exhibit high degrees of separation even when expanding this window to 1336.42 nm (Fig 7; *F* = 78.3, *p* = 1.11 ×10^−18^). In either of these cases, the likelihood of a False Positive is very low, with a probability of 0.002%, even with worst-case scenario prior odds of 2:10 (FPR = 0.008%). For the case of H against BCC this probability was calculated 1.25 ×10^−14^%, and 4.98 ×10^−14^ with prior probabilities of 2:10. From this perspective, these shorter wavelengths of the NIR spectrum can be considered of substantial importance in the identification of malignant skin samples in comparison with healthy skin.

Nevertheless, more substantial differences between H and SCC (F = 71.2, *p* = 4.05 ×10^−17^) with a 4.16 ×10^−13^ ∈ [1.04 ×10^−13^, 1.66 ×10^−12^]% probability of being a False Positive have also been detected at a longer wavelength, located between 1383.79 to 1483.25 nm. While this particular window coincides precisely with the region of worse differentiation between H and BCC (Fig 7), this window also coincides with a portion of the electromagnetic spectrum of particular importance for the discerning between both SCC and BCC samples. This latter window can be established between 1383.79 to 1454.83 nm (*F =* 51.2, *p* = 1.03 ×10^−12^), and has a 7.73 ×10^−09^ ∈ [1.93 ×10^−09^, 3.09 ×10^−08^]% probability of being a False Positive. In either case, this specific region of the electromagnetic spectrum coincides with an area of particular importance when considering water absorption rates and the saturation contents of skin. As can be seen in the respected signatures (Fig 6), this is the only region of the spectrum where SCC reflects more light than healthy skin (0.41%), while BCC absorbs the most amount of light (0.46%) in all cases.

Beyond this particular window, the NIR portion of the electromagnetic spectrum can be considered particularly useful for the differentiation between SCC and BCC samples (*F* = 28.6, *p* = 9.67 ×10^−08^), with two considerably important windows between 1109.06 and 1208.53 nm, as well as 1236.95 and 1506.94 nm (Fig 7). The probability that these windows are a False Positive remain relatively low; 4.25 ×10^−04^ ∈ [1.06 ×10^−04^, 1.70 ×10^−03^]%.

**Fig 8:**
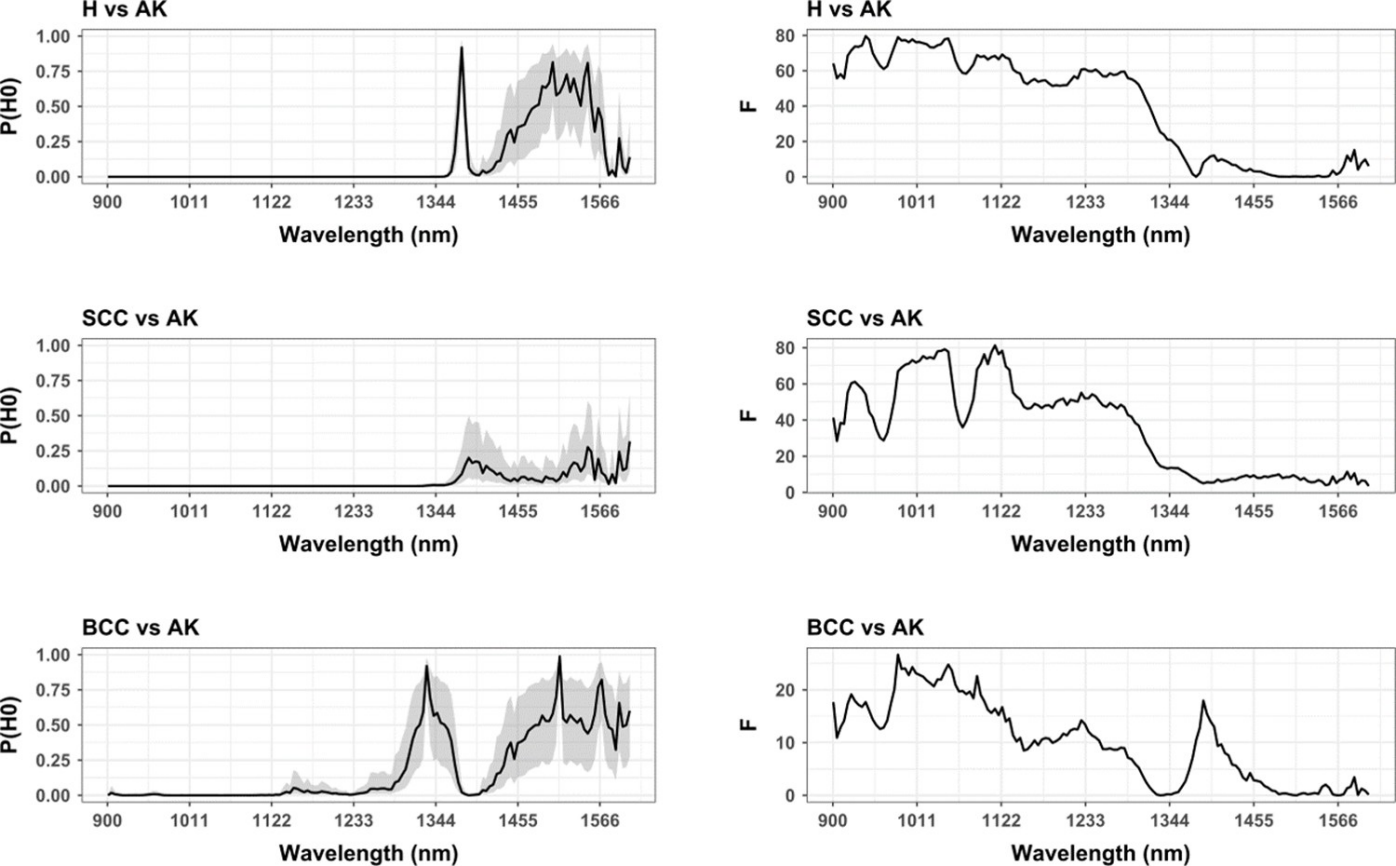
Univariate hypotheses test results comparing H, SCC and BCC with samples of AK across each of the hyperspectral bands using the Levene test for homoscedasticity. (Left panels) Probability of Null Hypothesis (P(H_0_)) curves, calibrated for each p-value using priors of 1:2 to mark the central tendency, while confidence intervals mark upper bounds using 2:10 prior probabilities in favour of H_0_, and lower bounds using 8:10 prior probabilities in favor our H_0_. (Right panels) Test statistic calculations for each of the corresponding hypothesis tests.

When considering the univariate differences between each of these samples and AK (Fig 8), the worst separation between samples can be found between BCC and AK, where the smallest window so far can be established between 981.18 and 1061.70 nm. The statistical differences between these samples in this region are generally low (*F* = 22.8, *p* = 1.88 ×10^−06^), however still present a low enough probability of being a False Positive to be considered valid (FPR = 0.007%). Even using prior probabilities of 2:10 that the *H*_*a*_ is correct, taking into consideration the possible bias or distortion induced sample size discrepancies, the probability of this observed separation being a False Positive is as low as 0.027%.

Finally, for both the case of discerning AK from H or SCC (Fig 8), considerably large windows have been identified from and 900.66 nm to 1345.89 nm for the case of H, and to 1322.21 nm for SCC. Based on the current samples, the separation between H and AK (*F* = 60.7, *p* = 8.03 ×10^−15^) is greater than the separation between SCC and AK (*F* = 50.9, *p* = 1.40 ×10^−12^), however, FPR values are still substantially low enough to identify valuable windows for the differentiation between these samples. In the case of H and AK, the probability of this conclusion being a Type I statistical error is 7.08 ×10^−11^ ∈ [1.77 ×10^−11^, 2.83 ×10^−10^]%. In the case of H and SCC, this probability has been calculated at 1.04 ×10^−08^ ∈ [2.60 ×10^−09^, 4.16 ×10^−08^]%.

### 3.3. Defining a final window

**Fig 9:**
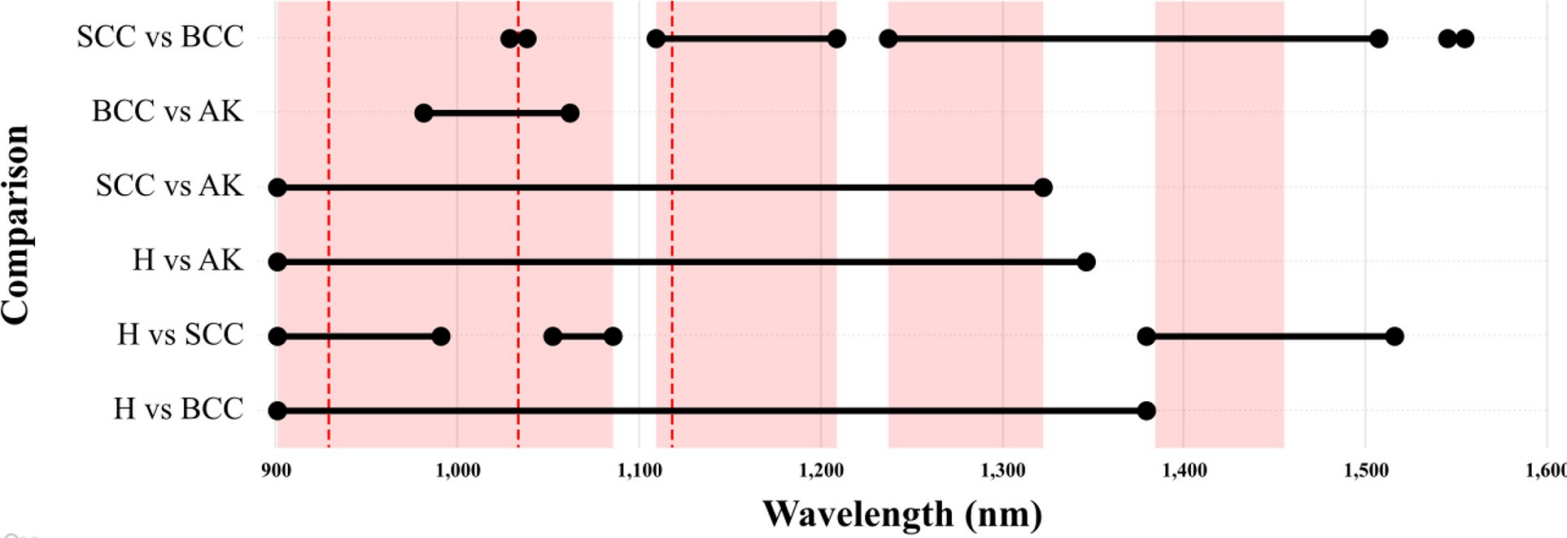
Optimally defined windows as established by the multiple statistical assessments presented within this study. Areas shaded in red mark the final window of interest captured over a total of 97 channels, including wavelengths between; 900.66 and 1085.38 nm, 1109.06 and 1208.53 nm, 1236.95 and 1322.21 nm, 1383.79 and 1454.83 nm. The three dotted lines identify the three channels of greatest differences for the optimal visualization of hyperspectral images using false colours; 929.08, 1033.28 and 1118.54 nm.

When comparing all the different results obtained throughout this study, a final window where the greatest differences appear to be located based on the present sample can be established between four different regions; 900.66 and 1085.38 nm, 1109.06 and 1208.53 nm, 1236.95 and 1322.21 nm, 1383.79 and 1454.83 nm (Fig 9). This window covers a total of 459.49 nm over 97 different bands.

Multivariate statistical testing across these regions reveal notable statistical differences between all samples, with Wilcox test results calculating *p* values of < 2.0 ×10^−16^ for all of the samples. This corresponds to a <1.97 ×10^−12^ ∈ [4.91 ×10^−13^, 7.86 ×10^−12^]% probability of this conclusion being a Type I statistical error.

When considering the top three optimal bands in these regions for the visualisation of hyperspectral images, the present study has found the wavelengths at 929.08, 1033.28 and 1118.54 nm to be particularly useful (Fig 9). Multivariate statistical testing of just these three wavelengths produce *p* values of < 5.5 ×10^−08^, thus resulting in a <2.50 ×10^−04^ ∈ [6.25 ×10^−05^, 9.99 ×10^−04^]% probability of being a Type I statistical error. From this perspective, these three channels may be the best means of visualizing hyperspectral images using false colour for the visual inspection of skin lesions.

**Fig 10:**
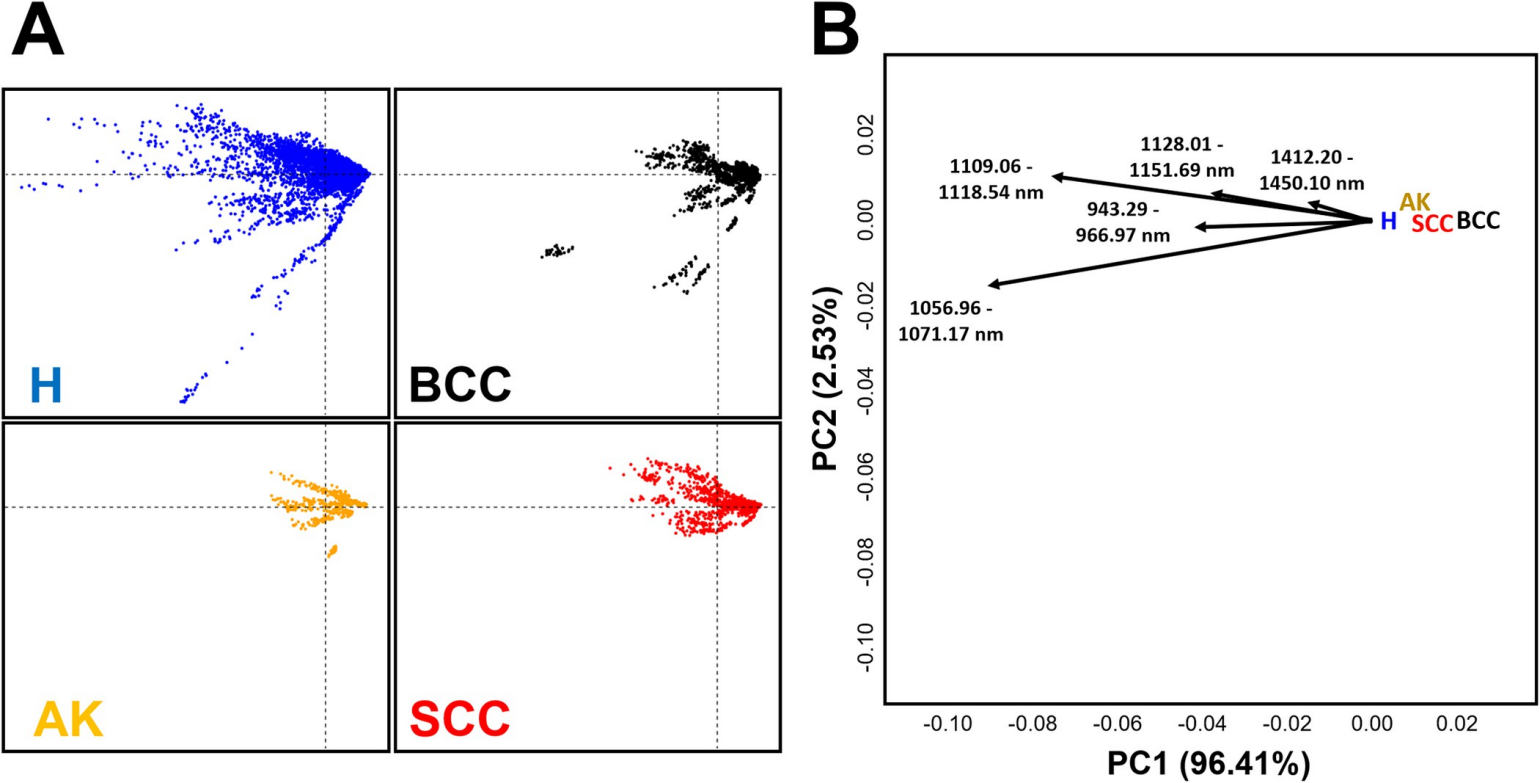
Nonlinear PCA (A) scatter and (B) bi-plots using a Spline Kernel for the multivariate analysis of the hyperspectral windows defined in the present study. (A) Distribution across the first PC scores of each of the samples. (B) Biplot presenting the robust central tendency in feature space of each of the sample as well as loading arrows indicating the most important variables and their directionality of the first 2 PC scores.

Finally multivariate ordination of the defined windows by means of k-PCA (Fig 10) reveal notable differences (*p* ≈ 0.001) between samples, corresponding to an FPR of 1.84 ∈ [0.47, 6.99]%. Sample distributions reveal healthy skin to present the greatest overall variability, followed by both NMSC groups and finally AK. Sample distributions across k-PCA feature space are mostly conditioned by reflectance values in the windows 1056.96 to 1118.54 nm, representing the trends that explain the vast majority of sample distributions (≈ 96.41% of the variance). Observing distributions across PC2 (2.53% of variance), it can be seen how windows between 1128.01–1151.69 nm and 1412.20–1450.10 nm also appear to condition the distribution of signatures in k-PCA. From this perspective, each of the NMSCs is seen to fall along this general axis, with the strongest overall trends observed for AK and SCC.

## 4. Discussion

HSI is a valuable, highly informative, and non-invasive tool for many biomedical imaging applications. The increased popularity of HSI research in the field of dermatology presents these types of sensors as a promising means of facilitating the screening, early diagnosis, and in-depth analysis of multiple types of skin cancer. At present, the most common means of performing these tasks typically relies on a dermatologist’s experience, or the use of more invasive procedures such as histopathological diagnoses. From this perspective, the ability of HSI to assess how light interacts with certain tissues provides a different perspective that may increase the precision and efficiency of simple visual inspections of skin [29,51].

Previous research has shown how the nature of tissue structure across the dermis and epidermis causes ‘significant’ changes to the scattering patterns between 1050 and 1400 nm, proving particularly useful in the differentiation between different NMSC samples [28]. Here we support these observations from the perspective of light reflectance patterns, extending this window slightly to 1455 nm. The NIR wavelengths included within this study have shown strong absorption rates for non-healthy samples across these regions. In most of these cases, such patterns can be attributed to water-saturation rates of the epidermis (Fig 1), corroborating observations made by multiple authors [22,28]. From this perspective, the current study reports absorption rates between 1383.79 and 1454.83 nm to be a useful biomarker for diagnostic purposes. Similarly, the region between 1109.06 and 1208.53 nm have also been noted to be a useful biomarker for discerning between NMSC classes, with BCC samples absorbing more light in this region than SCC.

In comparison with similar research carried out on other regions of the electromagnetic spectrum [25], here we have found a larger portion of the spectrum to present substantial differences between each of the samples using longer wavelengths. Using very similar statistical approaches, Courtenay et al., [25] reported False Positive Risks of up to 1.8% when differentiating between H and BCC in the yellow to red regions of the visible spectrum, while divergences between H and SCC were not detected at all using the Levene test. While the separation of H and SCC is still a challenge, the present study reports False Positive Risks lower than 0.0004% for these same comparisons.

When performing general assessments of data obtained in the visible spectrum and shorter wavelengths of IR light, it is important to point out the notable influence melanin has on the absorption of light by skin. This is especially relevant for wavelengths below 1100 nm [52]. While the degree of oxygenation in haemoglobin can also be detected in these regions [22,53], the natural variability of skin tissue across the population is a fundamental component to take into consideration for any type of analysis. From this perspective, the first window proposed here between 900.66 and 1085.38 nm is likely to be susceptible to change when considering samples from patients of differing origins. Beyond 1100 nm, however, the value of IR light in the study of skin samples becomes more apparent, as the influence skin colour has on reflectance values diminishes considerably [52]. Coupled with the ≈ 1370 μm greater penetration rate of IR light in skin [52], it can be seen how the use of NIR HSI may be more useful for the study of more intrinsic factors related to skin tissue composition and structure.

Beyond the advances presented within this study for the discerning between healthy and NMSC samples, the addition and in-depth analysis of AK can also be considered to be of great value. AK is often difficult to correctly diagnose [15,18,54], while high confusion rates with other types of lesions, in particular SCC, can hinder an effective treatment. In light of the present study, it is interesting to observe how AK samples are often found to lie between NMSC and healthy skin samples (Figs 6, 9 & 10). This is logical considering the progressive trajectory of AK to more serious skin conditions, such as SCC [10–12,18]. While it can be seen that the robust signature calculated for AK (Figs 6 and 9) presents similarities in certain regions of the spectrum with other samples (namely H), this is certainly due to the sample size. Nevertheless, regardless of sample size and balance issues, hypothesis testing regarding these samples still indicate a possible differentiation, while even in the worst of scenarios the present data only indicates a 0.10% probability that these conclusions are likely to be a False Positive.

Despite the advances presented by the introduction of HSI into dermatological imaging, it is also important to note that most diagnostic criteria in skin lesion research are based on a combination of variables, as opposed to a single variable alone [55,56]. At present, HSI provides a means of assessing a series of more ‘visual’ parameters regarding the colour and appearance of skin lesions. While IR light attains a greater depth of penetration, and thus offers information about both internal and external features undetectable to the naked eye, other variables such as symmetry, size, and border regularity, are also considered important diagnostic criteria. Considering the geometric resolution of these types of sensors, it would be interesting to see how multimodal datasets, combining both hyperspectral as well as morphological information [57], can aid in fine-tuning diagnostic tools.

## 5. Conclusions

The present study has employed the use of a push-broom NIR Hyperspectral Sensor with an *ad hoc* platform for the study of healthy skin, two different types of NMSCs, and AK. Robust statistical procedures have been able to highlight the value of this region of the electromagnetic spectrum, providing 4 windows for the differentiation between these samples. These windows include wavelengths between 900.66–1085.38 nm, 1109.06–1208.53 nm, 1236.95–1322.21 nm, and 1383.79–1454.83 nm, with an associated probability of <1% that our observations are a false positive based on the present data.

While research of this type is of great value to dermatology, future research into the hyperspectral characterisation of nevi should be considered a fundamental step towards the development of a more practical diagnostic tool. The inclusion of other problematic non-cancerous skin conditions, such as Seborrheic Keratosis, may also be an important addition to the datasets analysed. Similarly, it would also be interesting to see how lesion variability evolves with time, considering different stages of malignancy as well as growth. The inclusion of AK in the present study greatly helps some of these points, however, larger sample sizes are required before an in-depth characterisation and differentiation of these lesions can be performed.

Finally, the detection and classification of NMSC using deep learning and hyperspectral data should also be addressed in the future. Previous studies obtained promising results using Deep Learning models for pixel classification, even when using images with a less optimal range between 400 and 1000 nm [25]. From this perspective, it would be interesting to see how results change when considering the windows proposed by the current models.
